# 3-Hydroxypropionaldehyde production from crude glycerol by *Lactobacillus diolivorans* with enhanced glycerol uptake

**DOI:** 10.1186/s13068-017-0982-y

**Published:** 2017-12-07

**Authors:** Katharina Anna Lindlbauer, Hans Marx, Michael Sauer

**Affiliations:** 1 CD-Laboratory for Biotechnology of Glycerol, University of Natural Resources and Life Sciences, Vienna, Muthgasse 18, 1190 Vienna, Austria; 2 Department of Biotechnology, University of Natural Resources and Life Sciences, Vienna, Muthgasse 18, 1190 Vienna, Austria; 3 Austrian Centre of Industrial Biotechnology (ACIB GmbH), Muthgasse 11, 1190 Vienna, Austria

**Keywords:** Lactic acid bacteria, Biodiesel, Biorefinery, Crude glycerol, Glycerol utilization, Glycerol uptake, Glycerol dehydratase

## Abstract

**Background:**

In their quest for sustainable development and effective management of greenhouse gas emissions, our societies pursue a shift away from fossil-based resources towards renewable resources. With 95% of our current transportation energy being petroleum based, the application of alternative, carbon-neutral products—among them biodiesel—is inevitable. In order to enhance the cost structure of biodiesel biorefineries, the valorization of the crude glycerol waste stream into high-value platform chemicals is of major importance.

**Results:**

The purpose of this study is the production of 3-hydroxypropionaldehyde (3-HPA) from biodiesel-derived crude glycerol by *Lactobacillus diolivorans*. Particular focus is given on overcoming potential limitations of glycerol transport into the cell, in order to use the cells’ total glycerol dehydratase capability towards the formation of 3-HPA as the main product. Recombinant overexpression of the endogenous glycerol uptake facilitating protein PduF results in a significant increase of glycerol conversion by a factor of 1.3. Concomitantly, glycerol dehydratase activity increased from initially 1.70 ± 0.03 U/mg protein to 2.23 ± 0.11 U/mg protein. With this approach, an average productivity of 4.8 g_3-HPA_/(g_CDM_ h) yielding up to 35.9 g/L 3-HPA and 0.91 mol_3-HPA_/mol_Glycerol_ have been obtained.

**Conclusion:**

*Lactobacillus diolivorans* proves to be a valuable cell factory for the utilization of crude glycerol delivering high-value C3 chemicals like 3-HPA, 1,3-propanediol (1,3-PDO) and 3-hydroxypropionic acid (3-HP). Enhancing the glycerol influx into the cell by genetic engineering was successful paving the way towards the commercial production of 3-HPA.

**Electronic supplementary material:**

The online version of this article (10.1186/s13068-017-0982-y) contains supplementary material, which is available to authorized users.

## Background

Between 1970 and 2011, global greenhouse gas (GHG) emissions (particularly carbon dioxide) derived from fossil fuel combustion and industrial processes increased by 75% [1]. Key drivers for this dramatic increase in GHG can be related to alterations in population, structure of the economy, state of technology, and availability of fossil energy resources [2]. To counteract this trend, legal frameworks like the Paris Agreement aim at tackling global climate change, based on wide ranging changes and innovations for energy provision [3]. 60% of new power generation capacities are projected to be derived from renewables with the majority of them being competitive without any subsidies by 2040 [4]. This trend towards renewable energy technologies is also reflected in the growth of the global biofuels market—including biodiesel and bioethanol—from $148.5 billion in 2015 up to $169.6 billion until 2021 [5]. This performance is accompanied by an increasing availability of crude glycerol which is derived as a side stream from the transesterification process in amounts of 10% (w/w) of the total biodiesel production volume [6, 7]. High abundance, low price, and high degree of reduction make glycerol an attractive feedstock for biorefineries [8]. However, crude glycerol contains many impurities, therefore cost-intensive product purification and recovery measurements are required for commercial sale in technical quality. The potential revenue is quite low due to the currently low spot price of $0.79/kg for technical quality refined glycerol [9–12]. Therefore, the upgrade of crude glycerol to high-value chemicals is desirable. A concomitant production of fuels and chemicals in an integrated biorefinery, meets the environmental and economic goals simultaneously, due to high-value chemicals becoming the economic driver for the entire production plant [13, 14].

A product of interest is 3-hydroxypropionaldehyde (3-HPA) which is currently available as a specialty chemical at a minimum price of $270/g (95% purity, Atlantic Research Chemicals Ltd.) [15]. This metabolite is well known for its antimicrobial activity. A further market (albeit at significantly lower price) is addressed by its role as production intermediate for platform chemicals like 3-hydroxypropionic acid (3-HP), acrolein, or most importantly acrylic acid [16–18]. Chemical and biological approaches have been explored to convert glycerol into 3-HPA [6, 19]. The advantages of microbial conversion over conventional chemical production processes include higher specificity at moderate process conditions in regard to temperature and pressure, but also higher tolerance to impurities and the possibility to use variable feedstocks [20, 21]. Natural production of 3-HPA has been reported for species of *Bacillus, Citrobacter, Clostridium, Enterobacter, Klebsiella,* and *Lactobacillus* [18, 22, 23]. The microbial production is a simple one-step conversion process, catalyzed by the redox neutral action of glycerol dehydratase (GDHt) yielding 3-HPA by dehydration [24]. This conversion step is neither connected to the redox nor the energy metabolism of the cells. It is the first natural step of the metabolic pathways from glycerol to 1,3-PDO (electron sink) or to 3-HP (yielding one ATP and 2 electrons) [25]. Due to the high toxicity of 3-HPA, the metabolic pathways are tightly balanced in growing cells, to prevent any accumulation of the intermediate. Natural accumulation of 3-HPA has been reported to occur either due to an enzymatic imbalance, involving high glycerol dehydratase activity combined with low aldehyde dehydrogenase activity or in non-growing but metabolically active cells, also referred to as “resting cells.” Such cells turned out to be quite favorable for the targeted production of 3-HPA with minor accumulation of by-products [26, 27].

In this study, we are focusing on *Lactobacillus diolivorans* as microbial cell factory. The advantage of this organism is its ability to utilize both pharma grade, as well as crude glycerol without further processing steps, featuring no adverse impact of potential inhibitors on either growth or product yield. It has been shown that *L. diolivorans* is an excellent natural producer of 1,3-PDO and 3-HP, but the potential accumulation of 3-HPA has not been reported so far [28–30]. This study aims at a combinatorial approach of process and metabolic engineering to extend the product range of this cell factory towards the accumulation of 3-HPA. The main aspects include the evaluation of the glycerol uptake capabilities of *L. diolivorans* as well as the activity of glycerol dehydratase as both are key steps towards the formation of 3-HPA. The final aim is the establishment of an enhanced process for the production of 3-HPA from crude glycerol without application of any scavengers.

## Methods

### Bacterial strains and plasmids

*Lactobacillus diolivorans* LMG 19668 was used for all experiments in this study. The sequence of the glycerol facilitating protein PduF of *L. diolivorans* flanked by two BbsI restriction sites was ordered from IDT as part of a pUCIDT vector carrying a kanamycin resistance cassette. The expression of *pduF* was put under the control of the GAP promotor of *L. diolivorans* as well as the terminator of the chloramphenicol resistance cassette (TT_CAT) via GoldenMOCS [31], a modified form of Golden Gate cloning. This expression cassette was finally cloned into a vector carrying a replication origin of *L. diolivorans* (repA), as well as a kanamycin and erythromycin resistance (Additional file 1: Figure S1). In case of the empty vector (EV) control, the aforementioned vector was used without the introduction of an expression cassette for *pduF*. The construction of plasmids was performed in *E. coli DH10B* and the final propagation for the delivery of non-methylated DNA took place in *E. coli JM110*. Detailed information regarding the used strains and design of the plasmids is available in Additional file 1: Table S1 and Table S2.

### Transformation procedure

The transformation of plasmids was performed according to the protocol of Pflügl et al. [32] with some modifications which are described in brief. MRS medium “pH 5.7” supplemented with 2% (w/v) glucose was inoculated with a 1.5-mL cryo stock of *L. diolivorans* LMG 19668 and incubated overnight (30 °C, 180 rpm). An aliquot of this overnight culture was used to inoculate 500 mL MRS medium “pH 5.7” supplemented with 2% (w/v) glucose and 1% (w/v) glycine to an OD_600_ of 0.25 AU. After 3 h of incubation (30 °C, without shaking), cells were harvested by centrifugation (5 min, 4000*g*, 4 °C), washed four times with 0.3 M sucrose supplemented with 20 mM MgCl_2_ and once with 0.3 M sucrose. The cells were finally resuspended in an electroporation buffer (272 mM sucrose, 7 mM sodium phosphate, 0.5 mM MgCl_2_, pH 7.4). An 80 µL aliquot of cells was mixed with 20 µL DNA solution (3 µg) and electroporation was carried out at 2500 V, 200Ω, and 25 µF in an electroporation cuvette with 4-mm gap width. Immediately after, the cells were resuspended in 900 µL MRS medium “pH 5.7” supplemented with 2% (w/v) glucose, 0.3 M sucrose, and 20 mM MgCl_2_. This mix was transferred into a 1.5-mL Eppendorf tube, placed on a tube rack and positioned in a shaking incubator (30 °C, 180 rpm, 3 h). After this regeneration period, the cell suspension was plated on degassed MRS plate’s “pH 5.7” containing 2% (w/v) glucose and 2.5 µg/mL erythromycin and was incubated in an anaerobic jar at 30 °C for 3 days.

### Growth medium

The cultivations were performed in a two-step cultivation process. First of all, biomass was generated in a seed reactor containing a modified form of the MRS medium as described by De Man et al. [33] supplemented with 5 mg/L vitamin B_12_, 2.5 µg/mL erythromycin, 3% (w/v) glucose and either 1% (w/v) crude glycerol or 0.5% (w/v) 1,2-PDO. The second step, namely biotransformation, was performed in varying amounts of crude glycerol supplemented with 5 mg/L vitamin B_12_ and 2.5 µg/mL erythromycin. Crude glycerol used in this study was obtained from Universal Adsorbent & Chemicals Public Co. Ltd. Thailand and had an original concentration of 810 g/L glycerol. For the individual experiments, crude glycerol was used without further pretreatment and was accordingly diluted with water. The medium of the seed reactor was adjusted to pH 5.7 using 32% HCl, whereas the medium for biotransformation was adjusted to pH 7 using 4 M NaOH. Both media were supplemented with 1% (v/v) Struktol^®^ SB2121 dropwise to avoid foam formation, when required.

### Bioreactor cultivations

All bioreactor cultivations were performed in DASGIP^®^ parallel bioreactor systems (Eppendorf International) using three biological replicates—if not otherwise mentioned. For the seed reactors, an initial cultivation volume of 700 mL was inoculated to an OD_600_ of 0.2 AU (initial active biomass characterization experiments) or 0.4 AU (active biomass for biotransformation experiments) with 1.5% (v/v) inoculum from an exponentially growing preculture. These seed reactors were operated at a constant temperature of 30 °C, pH 5.7, 400 rpm agitation, and 2 sL/min N_2_-gassing in order to maintain anaerobic conditions. Throughout the cultivation process, pH 5.7 was maintained by the use of 12.5% NH_3_ as base supply.

### Cell harvest

Towards the end of the exponential growth phase, cells were harvested by centrifugation (4000*g*, 5 min, 20 °C). The pellet was washed with sterile water, centrifuged again and finally resuspended in sterile water accounting for 1.5% (v/v) of the original seed culture volume.

### Biotransformation

The cell fraction from the seed reactors was used as inoculum for the biotransformation process. Thereby the reactors were operated with a culture volume of 350 mL at a constant temperature of 30 °C, pH 7, 400 rpm agitation, and 2 sL/min N_2_-gassing. Throughout the cultivation process, pH 7 was maintained by the use of 12.5% NH_3_ as base supply.

### Measurement of cell growth

Biomass production was determined by measuring the optical density at 600 nm, and cell dry mass (CDM) was calculated by a correlation established by Pflügl et al. [28].

### Quantification of extracellular metabolites

Extracellular metabolites like d-glucose, glycerol, 3-HPA, 3-HP, 1,2-PDO, 1,3-PDO, lactic acid, acetic acid, and ethanol were determined by HPLC analysis (Shimadzu, Korneuburg, Austria) with an Aminex HPX-87H column (300 mm × 7.8 mm; Biorad) equipped with a Micro-Guard Cation H Cartridge (30 mm × 4.6 mm, Biorad). The column was operated at 60 °C and a flow rate of 0.6 mL/min with 0.004 M H_2_SO_4_ as mobile phase. A refraction index detector (RID-10A, Shimadzu, Korneuburg, Austria) and a UV–VIS photodiode array detector (SPD-M20A, Shimadzu, Korneuburg, Austria) were used for detection. We observed a tailing peak for 3-HPA in the RID detector which is overlapping with the peak of 1,3-PDO and therefore compromising the accurate quantification of this metabolite. As a result thereof, 3-HPA quantification was performed by using the UV detector at 210 nm [34] where the presence of small amounts of 1,3-PDO can be neglected due to its extremely low signal intensity (Additional file 2: Figure S2). Moreover, as the concentration of 1,3-PDO cannot exceed the levels of equimolar amounts of 3-HP, maximum levels of 0.84 g/L are expected in the presence of 1 g/L 3-HP.

### Cell breakage

Cells from the seed reactors or the biotransformation process were centrifuged (15,000*g*, 2 min, 4 °C), washed with degassed 20 mM potassium phosphate buffer (pH 8) and resuspended in the same buffer. Cell suspensions were subjected to physical cell disintegration by a Bead Beater (Fast-Prep-24TM, MP biomedicals, USA) at 6 m/s for 20 s (5 cycles). Broken cells were centrifuged (15,000*g*, 20 min, 4 °C) and the supernatant was collected in an anaerobic atmosphere to measure glycerol dehydratase activity [35] and protein concentration [36].

### Glycerol dehydratase activity assay

GDHt activity from crude cell extract was measured according to the protocol of Sankaranarayanan et al. [35]. The composition of substrate mixture was 20 mM potassium phosphate buffer (pH 8), 3 mM MgCl_2_, and 40 mM 1,2-PDO and coenzyme solution contained 15 µM vitamin B_12_, 0.15 mM NADH, and 1.5 mM ATP as well as 24 U/mL yADH as coupling enzyme.

## Results and discussion

### Evaluation of major bottlenecks attributed to 3-HPA formation in *L. diolivorans*

**Fig. 1:**
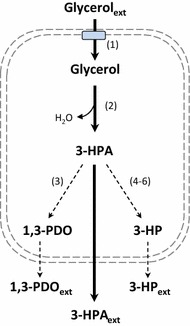
Utilization of glycerol in *L. diolivorans.* Illustration of the pathway for glycerol utilization in *L. diolivorans*. (1) Glycerol facilitator protein PduF; (2) vitamin B_12_-dependent glycerol dehydratase PduCDE; (3) 1,3-PDO oxidoreductase PduP; (4) PduP; (5) PduL; (6) PduW

The most crucial steps for the production of 3-HPA are the initial uptake of glycerol into the cell, followed by the conversion of the intracellular glycerol into 3-HPA by glycerol dehydratase (see Fig. 1). Members of the genus *Lactobacillus* are reported to accumulate 3-HPA extracellularly, ensuring a low, non-toxic level of intracellular 3-HPA [18, 37]—pointing to the fact that export is not limiting. Taking furthermore into account that the exporting transporters are currently not known, the following study is focusing primarily on limitations related to the glycerol uptake and further conversion to 3-HPA.

A first set of experiments aimed at the comparison of the GDHt activity with the actual rate of glycerol to 3-HPA conversion. To this end, batch cultures on MRS medium supplemented with 3% (w/v) glucose and 1% (w/v) glycerol were performed. Under this condition, 3-HPA is entirely converted into 1,3-PDO [28]. This conversion step proceeds in a fast and efficient manner within growing cells, ensuring that the concentration of toxic 3-HPA is kept low. We opted for this setup to gain insight into the potential of the biomass for the desired conversion without any interference of 3-HPA toxicity.

*Lactobacillus diolivorans* is not able to use glycerol as a sole carbon and energy source. This was confirmed by substrate utilization tests and C^13^ labeling experiments (data not shown). This characteristic is also reported for *Lactobacillus reuteri,* which lacks a dihydroxyacetone kinase required for channeling glycerol into the central carbon metabolism [24]. Genome analysis of *L. diolivorans* reveals that a bona-fide dihydroxyacetone kinase is also missing in this organism. There is a bioinformatic annotation for a “kinase to dihydroxyacetone kinase” (83 amino acids, NCBI Sequence ID: WP_057864359.1), which is rather short compared to the 356 amino acid long dihydroxyacetone kinase of other organisms like *K. pneumoniae* [38, 39] and therefore most probably not functional. This could be the reason why glycerol cannot serve as sole carbon and energy source in *L. diolivorans.*

Glycerol is converted via a one-step reaction towards the formation of 3-HPA in *L. diolivorans*. This reaction is catalyzed by a glycerol dehydratase, encoded by the gene cluster *pduCDE* [40]. As there is no side reaction diverting the flux of glycerol into biomass formation, the glycerol uptake rate is equal to the rate of glycerol conversion to 3-HPA. In addition to this, the potential of glycerol conversion based on the present GDHt activity can be derived via an in vitro enzymatic assay from crude cell extract as described by Sankaranarayanan et al. [35]. These two values are based on either cell dry mass—in case of glycerol uptake and conversion rates—or on measured protein content of the crude cell extract in case of enzymatically measured GDHt activity. Both values can be compared based on the assumption of bacterial protein content accounting for around 50% of CDM [35, 41, 42].

$${\text{Glycerol}}\;{\text{uptake}}\;{\text{rate}}\;q_{{\text{s}}} \;[{\text{g/g}}_{{{\text{CDM}}}}\,{\text{h}}] = {{\left( {\frac{{{\text{Glycerol}}_{{{\text{final}}}} - {\text{Glycerol}}_{{{\text{initial}}}} }}{{\Delta{\text{Time}}}}} \right)} \mathord{\left/ {\vphantom {{\left( {\frac{{{\text{Glycerol}}_{{{\text{final}}}} - {\text{Glycerol}}_{{{\text{initial}}}} }}{{\Delta \;{\text{Time}}}}} \right)} {{\text{CDM}}}}} \right. \kern-\nulldelimiterspace} {{\text{CDM}}}}$$

**Fig. 2:**
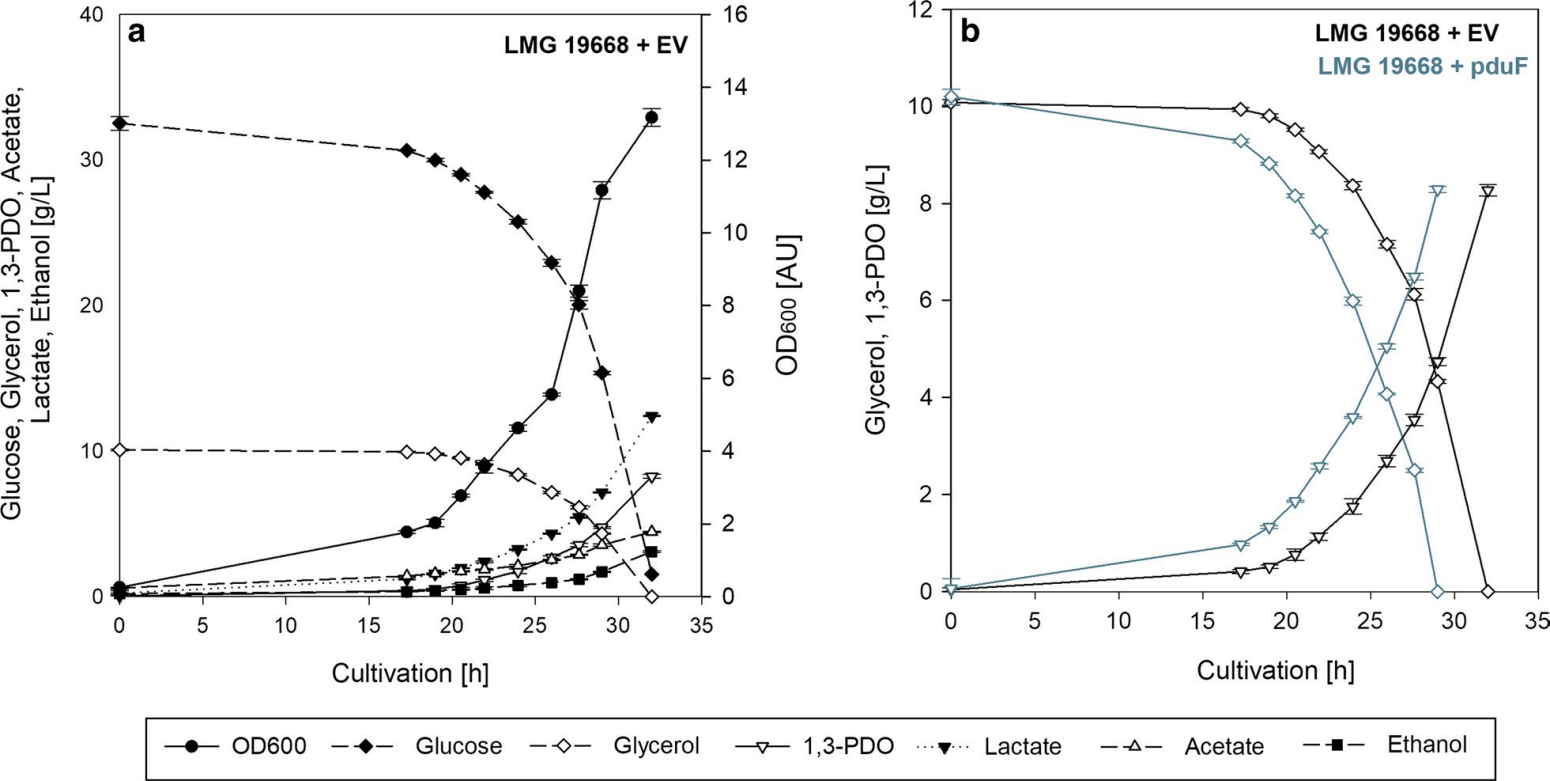
Batch cultivations of *L. diolivorans*. Illustration of batch cultivations on MRS-medium supplemented with glucose and glycerol of *L. diolivorans* LMG 19668 + EV (**a**) and comparison of this strain with *L. diolivorans* LMG 19668 + pduF strain in terms of glycerol uptake and 3-HPA formation capabilities (**b**)

**Fig. 3:**
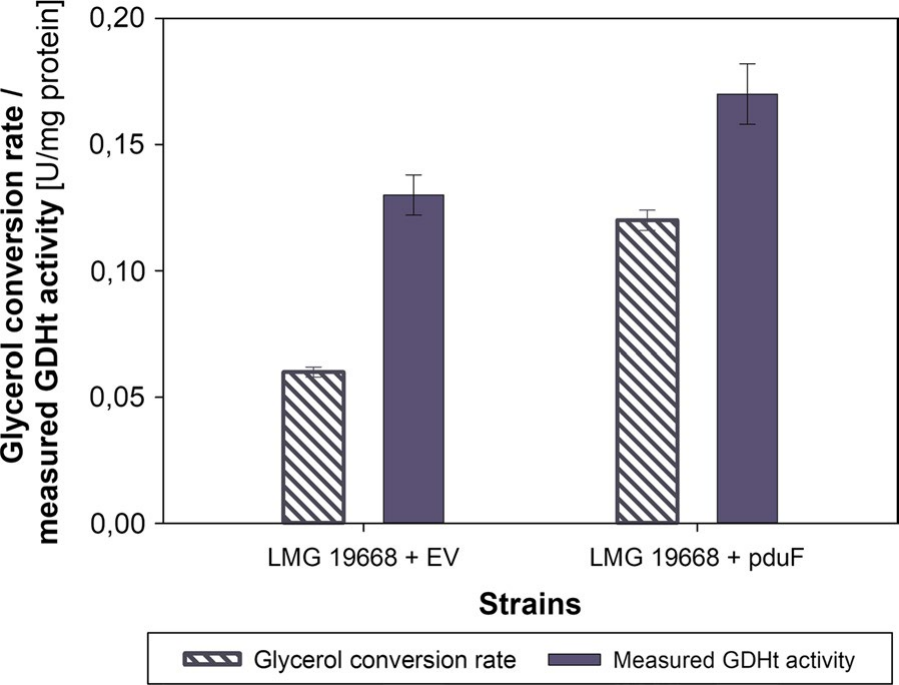
Illustration of glycerol conversion capabilities in *L. diolivorans.* Comparison of the glycerol conversion rate and enzymatically measured GDHt activity during batch cultivations on MRS supplemented with glucose and glycerol of *L. diolivorans* LMG 19668 + EV and *L. diolivorans* LMG 19668 + pduF

The performance of batch cultures of *L. diolivorans* LMG 19668 + EV is illustrated in Fig. 2a. Under this condition, glycerol is solely converted to 1,3-PDO as final product, without accumulation of 3-HPA or 3-HP in the fermentation broth [28]. The detailed analysis of these batch cultivations indicated that there is a significant discrepancy between the rate of glycerol conversion which equals the glycerol uptake rate. The required GDHt activity for this conversion can be calculated—it equals approximately 0.07 U/mg protein. The in vitro measured GDHt activity at the end of the exponential growth phase is 0.13 U/mg protein (Fig. 3). These results show that *L. diolivorans* LMG 19668 + EV uses just 54% of its actual glycerol dehydratase capabilities. This points to the fact that the glycerol dehydratase is not the limiting factor for glycerol conversion. In order to test, if the glycerol uptake is the limiting factor a genetic engineering approach was chosen aiming at the significant upregulation of glycerol uptake into the cell.

### Genetic engineering aiming at the upregulation of glycerol uptake

In order to enhance the glycerol uptake of *L. diolivorans*, the endogenous glycerol uptake facilitating protein PduF was overexpressed from a recombinant plasmid. This gene is part of the glycerol converting pdu operon of *L. diolivorans* and shows a high sequence similarity with glycerol uptake facilitator proteins for members of the species *Lactobacillus.* Moreover, there is a sequence similarity to the experimentally characterized glycerol facilitator GlpF from *E. coli,* which is responsible for the passive transport of small hydrophilic species like glycerol across the inner membrane [43]. Genome analysis also revealed that beside the *pduF* gene there are no further annotations for members of the major intrinsic protein (MIP) family (glycerol uptake facilitators, aquaporins), indicating the importance of this gene. In a baker’s yeast based complementation assay, we could confirm the capability of the *pduF* encoded protein to enhance glycerol uptake (data not shown).

In order to determine differences related to the uptake of glycerol between the strains *L. diolivorans* LMG 19668 + EV and *L. diolivorans* LMG 19668 + pduF, batch cultures on MRS medium supplemented with 3% (w/v) glucose and 1% (w/v) glycerol were performed and the actual GDHt activity was determined towards the end of the exponential growth phase. The overexpression of *pduF* leads to a significant increase of the average glycerol uptake rate by a factor of 1.3, from initially 0.21 g/(g_CDM_ h) to 0.27 g/(g_CDM_ h) (*q*_s, max_ = 0.35 g/(g_CDM_ h)). This points to the fact that the glycerol uptake has been limiting before (see Fig. 2b). Surprisingly, the measured GDHt activity in the *L. diolivorans* LMG 19668 + pduF strain is also increased, reaching a value of 0.17 U/mg protein at the end of the exponential growth phase and leaving again a gap between the potential for glycerol conversion based on measured GDHt activity and the actual rate of glycerol conversion (Fig. 3).

These results prove that the overexpression of *pduF* in *L. diolivorans* has a positive effect on the glycerol uptake rates and they indicate that overexpression of *pduF* leads to significantly higher GDHt activity values.

### Increased induction of glycerol dehydratase activity in *L. diolivorans*

Though GDHt featured significantly higher activity values in the strain overexpressing *pduF*, the overall measured GDHt activity of 0.17 U/mg protein is still low for an efficient 3-HPA production process. To further increase the activity of glycerol dehydratase, glycerol is substituted with 1,2-PDO as inducing agent in the growth medium. This has been reported to achieve significantly higher dehydratase activity values for *L. reuteri* [44].

For the generation of active biomass, batch cultures on MRS medium supplemented with 3% (w/v) glucose and 0.5% (w/v) 1,2-PDO were performed. It could be demonstrated that the addition of 1,2-PDO or glycerol leads to a similar growth behavior, though the supplementation with 1,2-PDO yields a significantly higher measured GDHt activity (0.85 U/mg protein or 1.11 U/mg protein for the strains *L. diolivorans* LMG 19668 + EV or *L. diolivorans* LMG 19668 + pduF, respectively). Also in this case, the *pduF* overexpressing strain has an enhanced activity compared to the empty vector control. Interestingly, the obtained activity is also significantly higher compared to the reported GDHt activity of 0.55 U/mg protein for *L. reuteri* induced by a comparable amount of 1,2-PDO (Table 1).

**Table 1:** Evaluation of GDHt activity in *Lactobacillus diolivorans*

	Glucose + Glycerol	Glucose + 1,2-PDO
*L. diolivorans LMG19668* + *EV*	56 mM/109 mM	56 mM/68 mM
	0.13 U/mg protein	0.85 U/mg protein
*L. diolivorans LMG19668* + *pduF*	56 mM/109 mM	56 mM/68 mM
	0.17 U/mg protein	1.11 U/mg protein
*L. reuteri 20016* [44]	15 mM/50 mM	15 mM/50 mM
	0.07 U/mg protein	0.55 U/mg protein

Comparison of enzymatically measured GDHt activities derived from cultivations on glucose supplemented with either glycerol or 1,2-PDO including the strains *L. diolivorans* LMG 19668 + EV, *L. diolivorans* LMG 19668 + pduF, and *L. reuteri*

### Bioconversion of glycerol with resting cells of *L. diolivorans*

For the production of 3-HPA, a two-step cultivation process consisting of the generation of active biomass, followed by a quick bioconversion of glycerol, is the most promising approach. This two-step process design has been reported to achieve the best results with other microbial cell factories in terms of high GDHt activity, high productivities, and suppression of by-product formation [27]. Therefore, bioconversion experiments using active cells of either *L. diolivorans* LMG 19668 + EV or *L. diolivorans* LMG 19668 + pduF with 1,2-PDO as inducing agent were performed on 2% (w/v) crude glycerol.

**Fig. 4:**
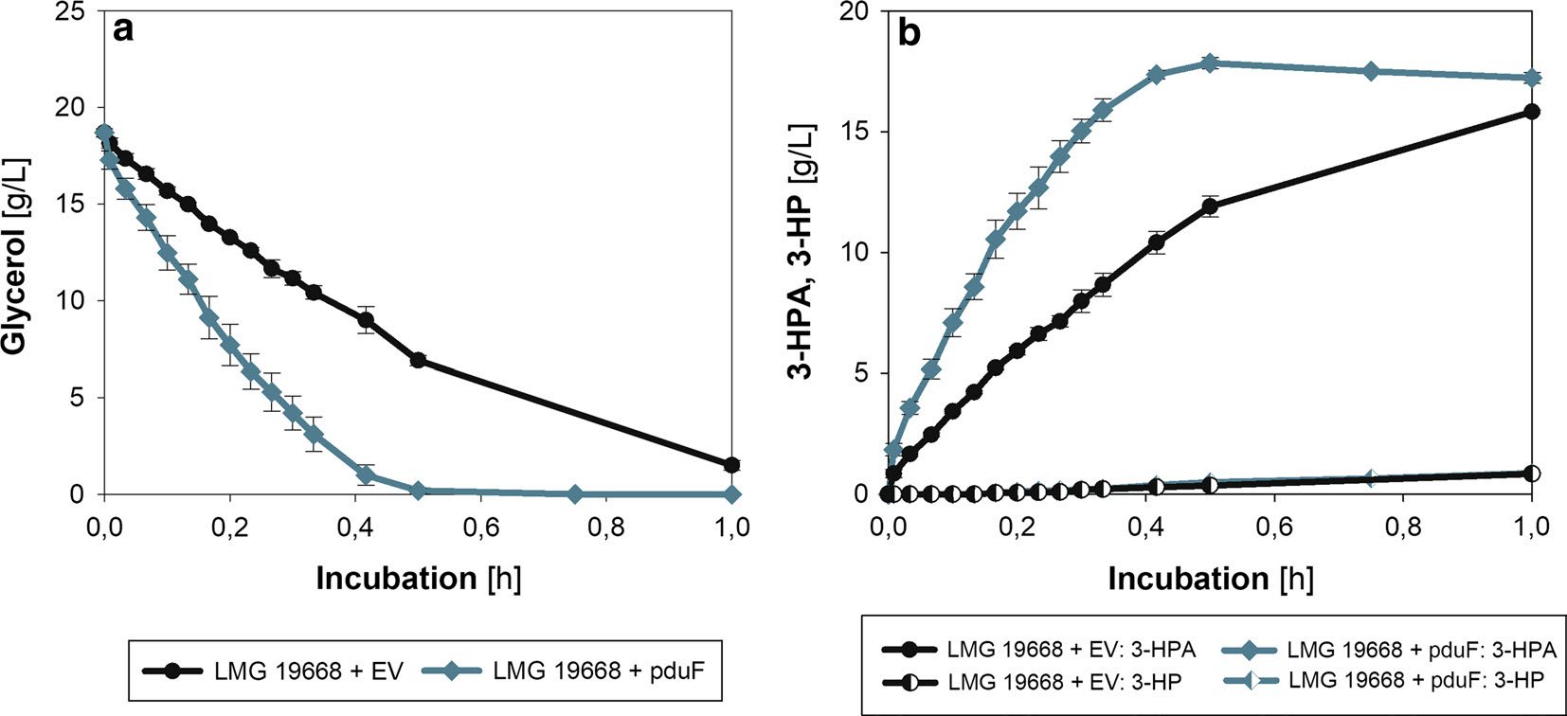
Bioconversion of glycerol by *L. diolivorans.* Illustration of glycerol consumption (**a**) and 3-HPA as well as 3-HP formation (**b**) during bioconversion of *L. diolivorans* LMG 19668 + EV (black) and *L. diolivorans* LMG 19668 + pduF (blue) on 2% glycerol. For a better illustration, levels of 1,3-PDO which are formed in equimolar amounts of 3-HP are not displayed in this figure. The active biomass for these bioconversion experiments was generated during batch cultivations on MRS-medium supplemented with 1,2-PDO

The bioconversion experiments showed that activated cells of *L. diolivorans* were excellent whole cell biocatalysts for the production of 3-HPA (Fig. 4). *L. diolivorans* LMG 19668 + pduF exhibited a significantly higher average glycerol uptake rate (*q*_S_ = 8.7 g/(g_CDM_ h), 0.5 h) as compared to the empty vector control (*q*_S_ = 4.2 g/(g_CDM_ h), 1 h). Within the first 0.5 h hardly any formation of 1,3-PDO and 3-HP was observed. Interestingly, by-product formation significantly increased after around 0.5 h for both strains, indicating potential time-dependent effects of increased 3-HPA levels in the fermentation broth. Possibly, rising energy requirements of the biocatalyst under stressful conditions lead to the slow dissipation of 3-HPA to 3-HP and 1,3-PDO. *L. diolivorans* LMG 19668 + pduF accumulated a maximum of 17.8 g/L 3-HPA with a molar yield of 99% and an average productivity of *q*_3-HPA_ = 5 g/(g_CDM_ h). In contrast to this, the LMG 19668 + EV strain exhibited a significantly higher level of by-product formation yielding a maximum of 15.8 g/L 3-HPA with a molar yield of 90% within 1 h. Additional File 3: Figure S3 illustrates the glycerol conversion rates during the 3-HPA production phase. The biocatalyst activity is gradually decreasing and leveling off around 0.5 U/mg protein upon complete consumption of glycerol. The apparent 3-HPA productivity has been compared with the measured GDHt activity after 10 min of the bioconversion reaction. As before, the in vitro measured GDHt activity is with 1.70 U/mg protein (*L. diolivorans* LMG 19668 + EV) and 2.23 U/mg protein (*L. diolivorans* LMG 19668 + pduF) significantly higher than the obtained glycerol conversion rates.

### Improving the performance of glycerol bioconversion in *L. diolivorans*

To produce industrially relevant levels of 3-HPA, overall titers need to be increased. Therefore, initial glycerol concentrations for the bioconversion process were raised from initially 2% (w/v) to 5% (w/v) crude glycerol.

**Fig. 5:**
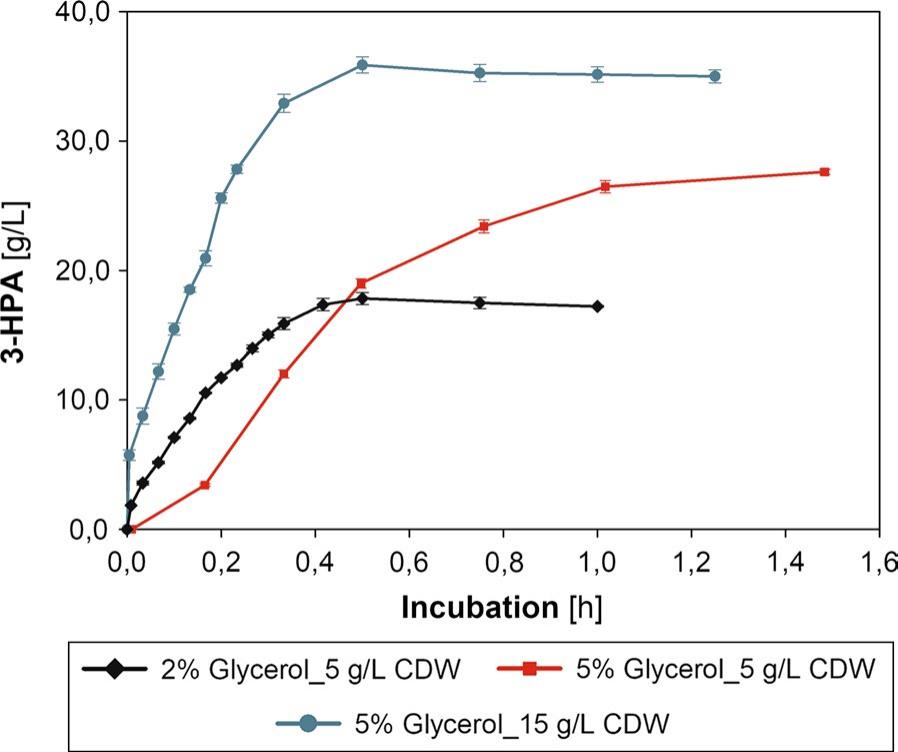
Effect of variations in initial glycerol concentration and amount of biocatalyst on production performance. Illustration of 3-HPA formation for *L. diolivorans* LMG 19668 + pduF for bioconversion processes starting with 5 g/L CDW biocatalyst and 2% (black) or 5% crude glycerol (red) as well as 15 g/L CDW biocatalyst and 5% crude glycerol (blue). The active biomass for these bioconversion experiments was generated during batch cultivations on MRS-medium supplemented with 1,2-PDO

This increase in initial glycerol concentration leads to an increase in final titers up to 28 g/L 3-HPA, however at the cost of productivity *q*_3-HPA_ = 3.5 g/g_CDM_ h and a low yield of 0.70 mol_3-HPA_/mol_Glycerol_ due to incomplete utilization of glycerol and increased by-product formation of around 1.3 and 1.2 g/L for 3-HP and 1,3-PDO, respectively (Fig. 5).

To overcome low productivities and ensure a complete utilization of glycerol, the amount of activated biocatalyst was increased from initially 5 g/L CDW up to 15 g/L CDW (Fig. 5). This increase in biocatalyst during bioconversions on 5% glycerol had a beneficiary effect on productivity, which reached 4.8 g/g_CDM_ h—similar to the performance on 2% (w/v) glycerol and with lower biocatalyst concentration. Furthermore, the overall titer could be improved to 36 g/L 3-HPA, corresponding to a molar yield of 91%. By-product formation using this approach was slightly increased up to 1.4 and 1.3 g/L for 3-HP and 1,3-PDO, respectively.

### Repeated batch bioconversions of *L. diolivorans*

A drawback of the two-step production process is the necessity of large amounts of active biomass. Biomass production has a significant impact on overall process costs. It also has a significant impact on real product yields because the carbon source required for the generation of active biomass must be taken into account from an industrial point of view. One option to circumvent this problem is the recycling of biomass at the end of the bioconversion process.

Therefore, cells from the end of a 3-HPA production process on 2% (w/v) glycerol were harvested, washed, and incubated in a fresh bioconversion media. However, 3-HPA accumulation started only after a significant lag phase of 15 min (data not shown). Therefore, also dehydratase activities measured after 10 min of incubation were very low (0.17 U/mg protein for *L. diolivorans* LMG 19668 + EV and 0.26 U/mg protein for *L. diolivorans* LMG 19668 + pduF, data not shown). Glycerol utilization stopped after 0.5 h reaching a final titer of only 9 g/L 3-HPA and a molar yield of 45% due to the incomplete utilization of glycerol. This indicates that the cells are severely damaged by the high 3-HPA concentrations to which they have been exposed during the first round of production. Hence, repeated batch bioconversion is not a suitable strategy for decreasing the amount of required active biomass without any protection of the biocatalyst.

### Comparison of the bioconversion of glycerol to 3-HPA by *L. diolivorans* to other cell factories

To our knowledge, the best values for biotechnological production of 3-HPA have been obtained with *L. reuteri*. Results obtained from *L. reuteri* cells activated with glycerol show that this organism is capable of producing up to 17.4 g/L 3-HPA from 36.8 g/L glycerol with a molar yield of 62% [45]. Decreasing the glycerol concentration to 20 g/L leads to an improved molar yield of 85% on the expense of the final 3-HPA concentration (12.6 g/L) [46, 47]. 1,2-PDO-activated cells of *L. diolivorans*—in comparison—accumulate 17.8 g/L 3-HPA from 2% crude glycerol with a molar yield of 99%.

A major point of hindrance for microbial production of 3-HPA is its toxicity, which has a serious negative impact on cell viability and pathway enzyme activity [35, 48]. One approach for overcoming these limitations is the protection of the biocatalyst by the application of scavengers like semicarbazide or carbohydrazide which are binding 3-HPA in form of hydrazone complexes and thereby allowing the accumulation of higher 3-HPA concentrations in the fermentation broth. Bioconversion using 1,2-PDO activated cells of *L. reuteri* in combination with semicarbazide as scavenger results in the production of 26 g/L 3-HPA from 5% glycerol with a productivity of 1.7 g/(g_CDM_ h) [24]. *L. diolivorans* cells activated by 1,2-PDO reach similar titers however with a significantly higher productivity of 3.5 g/(g_CDM_ h) in the absence of any scavengers—indicating superior biocatalyst properties under the tested conditions. Currently, *L. reuteri* features with the production of up to 150 g/L 3-HPA and a productivity of 10.7 g/L h the best 3-HPA production process [49]. However, the costs of scavengers and the increased costs for purification need to be taken into account.

Among other bacteria, *Klebsiella pneumoniae* is an alternative production host for 3-HPA. Its ability to use glycerol as a sole carbon source leads to a significant loss of substrate for biomass formation in case of growing cells. In a bioconversion process including scavengers, final titers of 46 g/L of 3-HPA with molar yields up to 80% are reported for this organism [50]. 3-HPA production within a fed-batch process featuring high scavenger concentrations and successive addition of active biomass was reported to achieve yields up to 97% (not taking the formation of biomass into account) thereby reaching the highest reported final titer of 54 g/L 3-HPA [51]. However, *K. pneumoniae* is considered a human pathogen, a characteristic which limits its industrial applications.

Another promising candidate for 3-HPA production is *Citrobacter freundii*. The highest concentrations of 3-HPA are reported for the *C. freundii* mutant strain HPAO-1 by obtaining a yield of 92% from 5% glycerol in the presence of semicarbazide hydrochloride [52].

## Conclusion

**Fig. 6:**
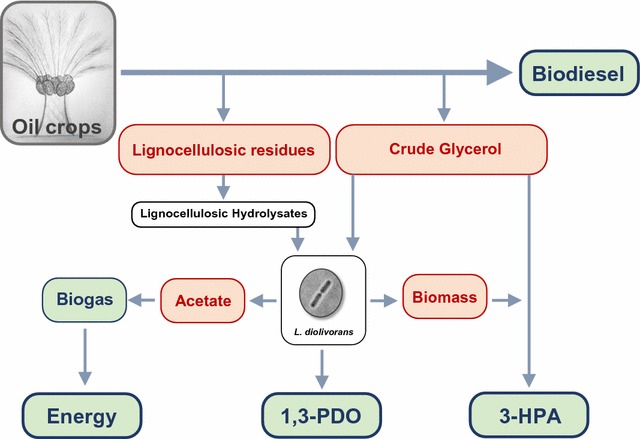
Integrated biorefinery concept for biodiesel production. Schematic illustration of a potential integrated biorefinery concept for biodiesel production including the valorization of side streams (red) towards the production of value-added chemicals and energy (green)

This study demonstrates for the first time the potential of *L. diolivorans* as a valuable cell factory for the valorization of crude glycerol towards 3-HPA extending the C3 product range from 1,3-PDO and 3-HP towards platform chemicals like acrolein and acrylic acid. The combinatorial approach of process and metabolic engineering enabled to overcome bottlenecks associated with glycerol uptake and significantly upregulated the activity of glycerol dehydratase. Based on our results, we propose an integrated biodiesel biorefinery concept (Fig. 6) where crude glycerol is upgraded in a first step into 1,3-PDO [28–30]. It could be demonstrated in previous studies that *L. diolivorans* is a promising cell factory for the production of 1,3-PDO from crude glycerol achieving maximum titers of 92 g/L 1,3-PDO with a molar yield up to 94% [30]. In order to improve the economic viability of the overall biorefinery concept, the valorization of side streams (beside crude glycerol) into new product and value streams is of major importance. The biomass obtained from the production of 1,3-PDO (0.09 ton CDM/ton 1,3-PDO) can be employed within a next process step upgrading crude glycerol into 3-HPA, which is another product of interest. Considering a 3-HPA production process as described in this study, maximum titers of 36 g/L 3-HPA with a molar yield of 91% can be obtained using *L. diolivorans* as microbial cell factory. The biomass obtained for the production of 1 ton of 1,3-PDO will allow the production of 0.21 tons of 3-HPA. A continuous supply of biomass originating from 1,3-PDO production eliminates the need for scavengers during 3-HPA production to protect the biomass. Moreover, the carbon-rich waste stream—mainly acetic acid—from the first process step can be used as input for biogas production. Acetic acid yields an energy output of 1300 kWh as methane if fed into a classical biogas fermentation. This concept is depicted in Fig. 6.

1,3-PDO as well as 3-HPA represent both interesting platform chemicals—the former as precursor chemical for the production of polyesters like polytrimethylene terephthalate (PTT) [53] and the latter one for the production of acrolein [54]. Bioprocesses for the production of both compounds from glycerol have been suggested in the literature. Among other lactic acid bacteria, *L. reuteri* has been reported to achieve 1,3-PDO productivities and yields comparable to *L. diolivorans*, though the maximum titers are with 65 g/L 1,3-PDO significantly lower [55]. For 3-HPA production with *L. reuteri*, a titer of 150 g/L has been reported—however, using scavengers to protect the cells [49]. The best 1,3-PDO process described to date is based on a recombinant *E. coli* strain within a fed-batch cultivation process with titers of 135 g/L 1,3-PDO, a productivity of 3.5 g/(Lh), and a weight yield of 51% based on corn-derived glucose [56]. The productivity is excellent; however, glucose as carbon source appears undesirable in context with our proposed biodiesel biorefinery concept. The significance of our proposed concept is the beneficial effect which can be derived by combining the two *L. diolivorans*-based production processes for 1,3-PDO and 3-HPA and integrating them into a biodiesel refinery. This results in a maximum valorization of the crude glycerol side stream into more profitable biochemicals. Furthermore, it significantly enhances the economics of 3-HPA production by eliminating the need for additional seed reactors containing cost-intensive MRS-medium for biomass generation or the application of scavengers for biomass recycling. In addition to this, the setup is also beneficiary for the step of 1,3-PDO production as the both major side streams (acetic acid, biomass) are continuously used in a next step. To conclude, this approach demonstrates an opportunity for creating an integrated oil plant-based biorefinery, producing biodiesel, 1,3-PDO, 3-HPA, and a significant amount of energy in form of biogas.

## Additional files

**Additional file 1: Figure S1.** *L. diolivorans* LMG 19668 + pduF. Schematic illustration of the BB3_AB_pGAP_pduF_TT CAT plasmid of *L. diolivorans* LMG 19668 + pduF. The BB3 linker includes an erythromycin resistance cassette from pSIP409 (ErmB) and kanamycin resistance cassette from pSTBLUE (Kan), the origin of replication from *L. diolivorans* (repA) and the origin of replication for *E. coli* from pUC19 (pUC_origin). The expression cassette includes the promoter of the glyceraldehyde 3-phosphate dehydrogenase (pGAP) from *L. diolivorans*, the terminator of the chloramphenicol resistance cassette (TT_CAT) from pC194 as well as *pduF* from *L. diolivorans* as the gene of interest. **Table S1**. Overview of strains and plasmids. **Table S2**. Primers used for PCR reactions.

**Additional file 2: Figure S2.** HPLC chromatograms. Illustration of a section of the peak pattern of the 5 g/L standard solutions of 3-HPA, 1,3-PDO and 3-HP (A, C) as well as the combination of these standards with the peak pattern of samples derived from bioconversion experiments (B, D) by using the RID detector (A, B) or the UV-VIS detector at 210 nm (C, D).

**Additional file 3: Figure S3.** Glycerol conversion rates during bioconversion. Illustration of the glycerol conversion rates of the strains *L. diolivorans* LMG 19668 + EV (black) and *L. diolivorans* LMG 19668 + pduF (blue) during bioconversion on 2% glycerol.

## Abbreviations

1,3-PDO: 1,3-propanediol
3-HP: 3-hydroxypropionic acid
3-HPA: 3-hydroxypropionaldehyde
GDHt: glycerol dehydratase
GHG: greenhouse gas emissions
MIP: major intrinsic proteins
PTT: polytrimethylene terephthalate
yADH: yeast alcohol dehydrogenase
