# Supplementary material for: 3-Hydroxypropionaldehyde production from crude glycerol by Lactobacillus diolivorans with enhanced glycerol uptake
Source: Biotechnol Biofuels. 2017 Dec 7;10:295. doi: 10.1186/s13068-017-0982-y (PMC5719546; doi:10.1186/s13068-017-0982-y)
Supplement: Supplementary file 1 — Additional file 1: Figure S1. L. diolivorans LMG 19668 + pduF. Schematic illustration of the BB3_AB_pGAP_pduF_TT CAT plasmid of L. diolivorans LMG 19668 + pduF. The BB3 linker includes an erythromycin resistance cassette from pSIP409 (ErmB) and kanamycin resistance cassette from pSTBLUE (Kan), the origin of replication from L. diolivorans (repA) and the origin of replication for E. coli from pUC19 (pUC_origin). The expression cassette includes the promoter of the glyceraldehyde 3-phosphate dehydrogenase (pGAP) from L. diolivorans, the terminator of the chloramphenicol resistance cassette (TT_CAT) from pC194 as well as pduF from L. diolivorans as the gene of interest. Table S1. Overview of strains and plasmids. Table S2. Primers used for PCR reactions. [file 13068_2017_982_MOESM1_ESM.docx]

**Additional file 1**


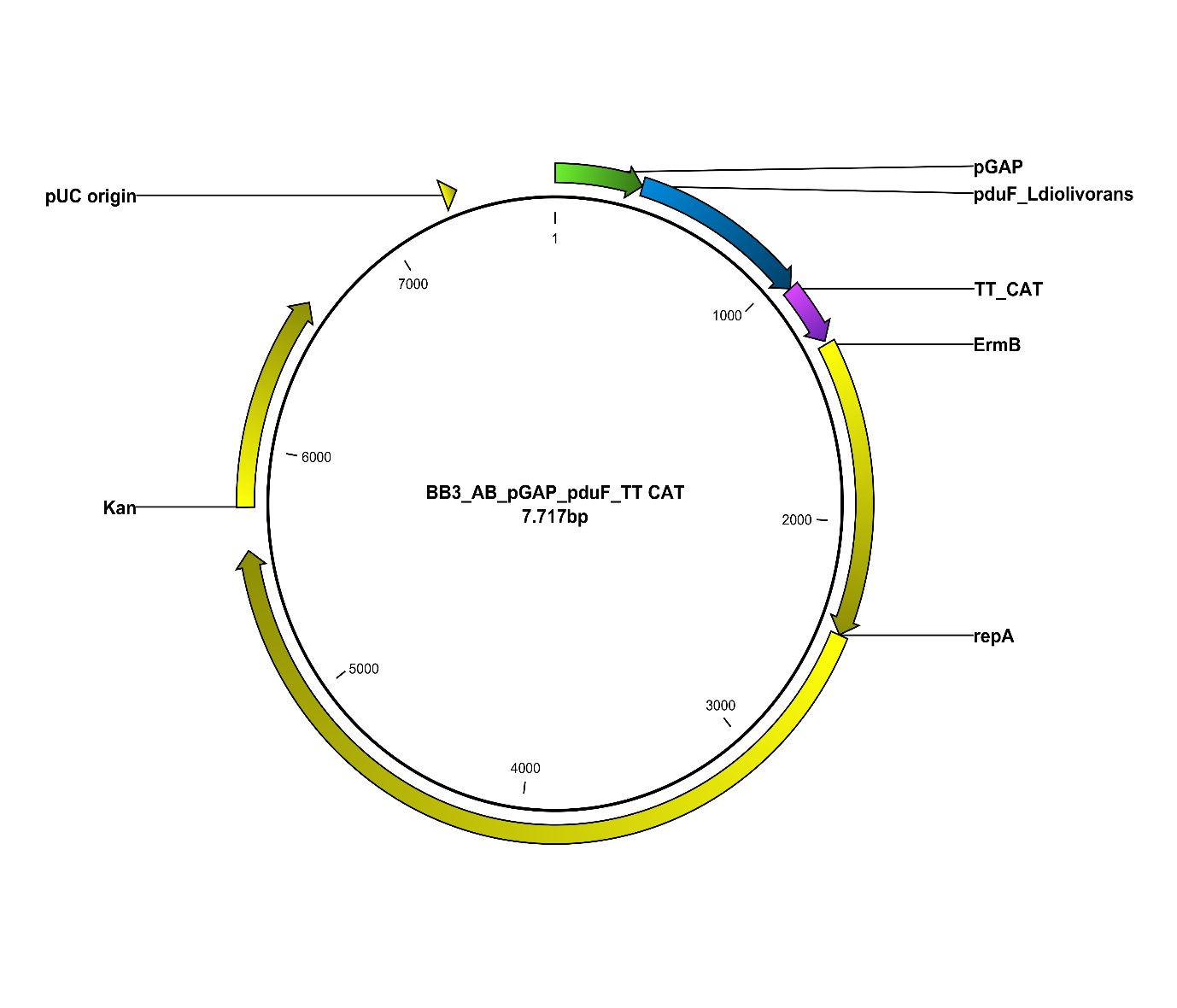


**Figure S1 L. diolivorans LMG 19668 + pduF**

Schematic illustration of the BB3_AB_pGAP_pduF_TT CAT plasmid of L. diolivorans LMG 19668 + pduF. The BB3 linker includes an erythromycin resistance cassette from pSIP409 (ErmB) and kanamycin resistance cassette from pSTBLUE (Kan), the origin of replication from L. diolivorans (repA) and the origin of replication for E. coli from pUC19 (pUC_origin). The expression cassette includes the promoter of the glyceraldehyde 3-phosphate dehydrogenase (pGAP) from L. diolivorans, the terminator of the chloramphenicol resistance cassette (TT_CAT) from pC194 as well as pduF from L. diolivorans as the gene of interest.

**Table S1 Overview of strains and plasmids**

| **Strain and plasmid** | **Description** |
| --- | --- |
| **Strains** |  |
| *L. diolivorans* LMG 19668 + EV | Expression host harboring an empty vector plasmid  (BB3_AB) |
| *L. diolivorans* LMG 19668 + pduF | Expression host harboring the pduF expression cassette  (BB3_AB_pGAP_pduF_TT CAT) |
| *E. coli DH10B* | F- *mcr*A Δ(*mrr*-*hsd*RMS-*mcr*BC) φ80*lac*ZΔM15 Δ*lac*X74 *rec*A1 *end*A1 *ara*D139 Δ (*ara*, *leu*)7697 *gal*U *gal*K λ- *rps*L *nup*G /pMON14272 / pMON7124  (Thermo Fischer Scientific, No. 18297010) |
| *E. coli JM110* | F'[traD36 proA+ proB+ lacIq delta(lacZ)M15] dam dcm supE44 hsdR17 thi leu thr rpsL lacY galK galT ara tonA tsx delta(lacproAB) lambda- (DSM, No. 11539) |
| **Plasmids** |  |
| BB1_L_12_syn_BsaI | Linker to construct BB1 from PCR product with FS1 and FS2, kan^r^ [[31](#_ENREF_31)] Addgene reference: 89914 |
| BB1_L_34_syn_BsaI | Linker to construct BB1 from PCR product with FS3 and FS4, kan^r^ [[31](#_ENREF_31)] Addgene reference: 89916 |
| BB1_P_12_ldi_pGAP | BB1 with GAP promoter of *L. diolivorans,* kan^r^ |
| BB1_T_34_syn_TT CAT | BB1 with Terminator of chloramphenicol resistance cassette, kan^r^ |
| pUCIDT_Kan_pduF | pUCIDT vector with pduF from *L. diolivorans,* kan^r^ |
| BB2_L_AB_syn_BbsI | BB2 Linker containing between FS1 and FS2 the BB3 linker with FSA and FSB, amp^r^ [[31](#_ENREF_31)] Addgene reference: 89917 |
| BB2_E_AB_pGAP_pduF_TT CAT | BB2 Linker containing the expression cassette for pduF  (GAP promoter, pduF and TT_CAT), amp^r^ |
| BB3_AB | BB3 Linker with FSA and FSB, kan^r^, ermb^r^ |
| BB3_AB_pGAP_pduF_TT CAT | BB3 Linker containing the expression cassette for pduF  (GAP_Pro, pduF and TT_CAT), kan^r^, ermb^r^ |

**Table S2 Primers used for PCR reactions**

| **Name** | **Function** | **Template** | **Sequence** |
| --- | --- | --- | --- |
| FS1_pGAP_FS2_FW | PCR fragment: GAP promoter from *L. diolivorans* with FS1 and FS2 | The central template was the pSHM plasmid as described by Pflügl et al. [[32](#_ENREF_32)] | GATCGAAGACGCGGAGGAAACATCATTTAATAATGCGCTC |
| FS1_pGAP_FS2_BW |  |  | GATCGAAGACGCCATGACTAAGTTTTCCTCCTTTAGGAAAT |
| FS3_TT CAT_FS4_FW | PCR fragment: terminator with FS3 and FS4 |  | GATCGAAGACGCGCTTTATGAGATAATGCCGACTGTAC |
| FS3_TT CAT_FS4_BW |  |  | GATCGAAGACGCAGCGGTCGGCATAGCGTGAG |
| FS2_ErmB_FS3_FW | PCR fragment for BB3 Linker: ErmB with FS2 and FS3 |  | GATCGAAGACGCCATGCCTTAGAAGCAAACTTAGAGTGT |
| FS2_ErmB_FS3_BW |  |  | GATCGAAGACGCAAGCCTCCATTCCCTTTAGTAACGTG |
| FS3_repA_FS4_FW | PCR fragment for BB3 Linker: repA with FS3 and FS4 |  | GATCGAAGACGCGCTTGAATTAACGAGTTACGCAAAG |
| FS3_repA_FS4_BW |  |  | GATCGAAGACGCAGCGTCTTTAGCGTCTTTGAACTCG |
| FS4_Kan_ori_FS1_FW | PCR fragment for BB3 Linker: kan_ori with FS4 and FS1 |  | GATCGAAGACGCCGCTGCGGCCGCCGTAACTGTCAGACCAAGTTTAC |
| FS4_Kan_ori_FS1_BW |  |  | GATCGAAGACGCCTCCGCGGCCGCGTTTCCTGTGTGAAATTGTTATCC |
