# Supplementary material for: 3-Hydroxypropionaldehyde production from crude glycerol by Lactobacillus diolivorans with enhanced glycerol uptake
Source: Biotechnol Biofuels. 2017 Dec 7;10:295. doi: 10.1186/s13068-017-0982-y (PMC5719546; doi:10.1186/s13068-017-0982-y)
Supplement: Supplementary file 2 — Additional file 2: Figure S2. HPLC chromatograms. Illustration of a section of the peak pattern of the 5 g/L standard solutions of 3-HPA, 1,3-PDO and 3-HP (A, C) as well as the combination of these standards with the peak pattern of samples derived from bioconversion experiments (B, D) by using the RID detector (A, B) or the UV-VIS detector at 210 nm (C, D). [file 13068_2017_982_MOESM2_ESM.docx]

**Additional file 2**

**Figure S2 HPLC chromatograms**

Illustration of a section of the peak pattern of the 5 g/L standard solutions of 3-HPA, 1,3-PDO and 3-HP (A, C) as well as the combination of these standards with the peak pattern of samples derived from bioconversion experiments (B, D) by using the RID detector (A, B) or the UV-VIS detector at 210nm (C, D).
