# Supplementary material for: 3-Hydroxypropionaldehyde production from crude glycerol by Lactobacillus diolivorans with enhanced glycerol uptake
Source: Biotechnol Biofuels. 2017 Dec 7;10:295. doi: 10.1186/s13068-017-0982-y (PMC5719546; doi:10.1186/s13068-017-0982-y)
Supplement: Supplementary file 3 — Additional file 3: Figure S3. Glycerol conversion rates during bioconversion. Illustration of the glycerol conversion rates of the strains L. diolivorans LMG 19668 + EV (black) and L. diolivorans LMG 19668 + pduF (blue) during bioconversion on 2% glycerol. [file 13068_2017_982_MOESM3_ESM.docx]

**Additional file 3**


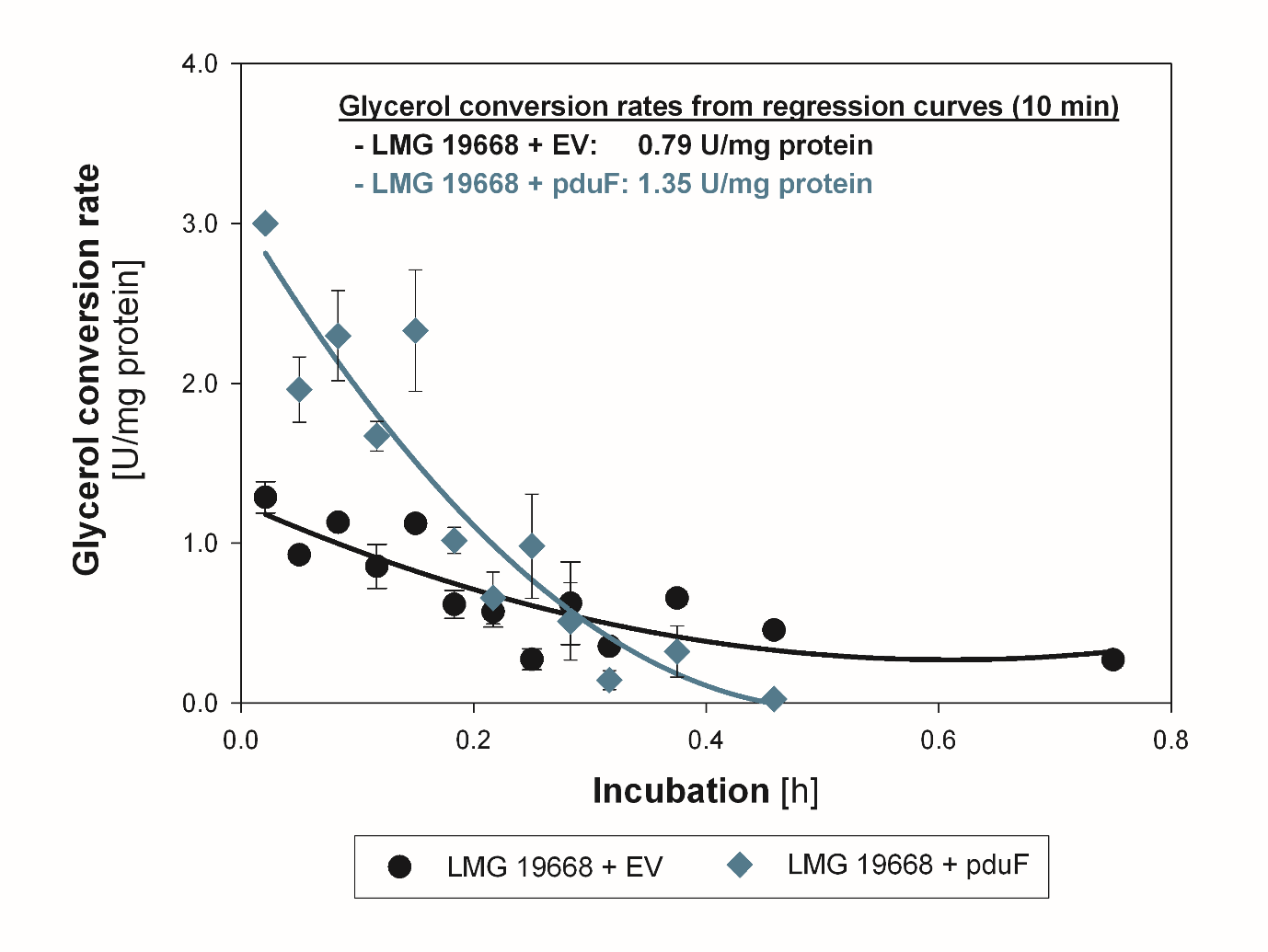


**Figure S3 Glycerol conversion rates during bioconversion**
Illustration of the glycerol conversion rates of the strains L. diolivorans LMG 19668 + EV (black) and L. diolivorans LMG 19668 + pduF (blue) during bioconversion on 2% glycerol.
